# Supplementary material for: Achievement of the Targets of the 20-Year Infancy-Onset Dietary Intervention—Association with Metabolic Profile from Childhood to Adulthood
Source: Nutrients. 2021 Feb 6;13(2):533. doi: 10.3390/nu13020533 (PMC7915301; doi:10.3390/nu13020533)
Supplement: Supplementary file 1 [file nutrients-13-00533-s001.pdf]

Figure S1.

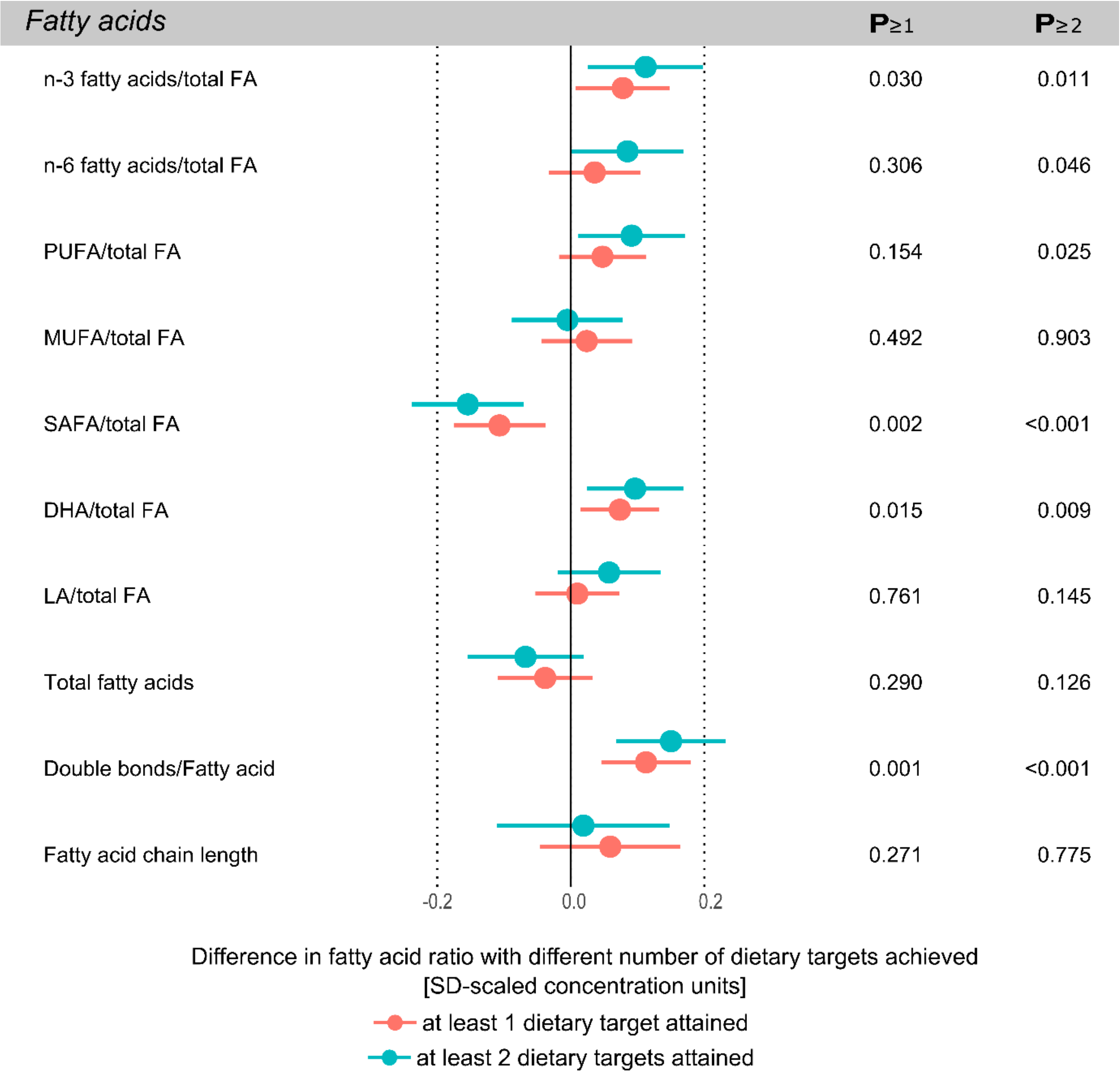

Figure 4. Differences in circulating serum fatty acids between achieving dietary targets compared to achieving none of the dietary targets when dietary target score includes a cholesterol target. Effect estimates are standard deviation scaled differences between achieving zero dietary targets with respect to achieving ≥1 (red) or ≥2 (blue) of the targets. Error bars indicate 95% confidence intervals. Metabolic measures are from pooled analyses across the 6 time-points. n-3 fatty acids/total FA denotes the ratio of omega-3 fatty acids to total fatty acids; PUFA, polyunsaturated fatty acids; MUFA, monounsaturated fatty acids; SAFA, saturated fatty acids; DHA, docosahexaenoic acid; LA, linoleic acid.

Figure S2.

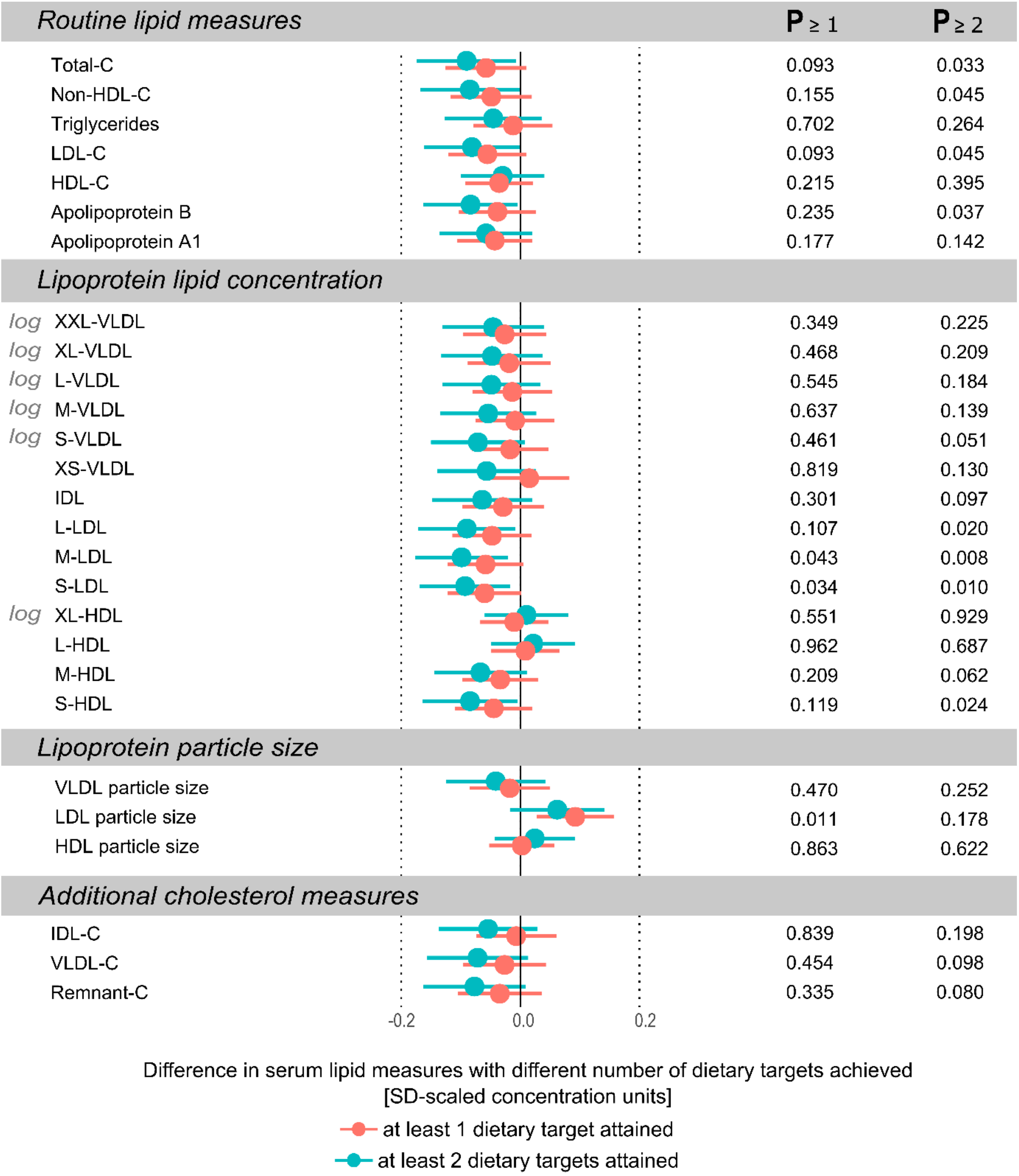

Figure 5. Differences in serum lipid measures between achieving dietary targets compared to achieving none of the dietary targets when dietary target score includes a cholesterol target. Effect estimates are standard deviation scaled differences between achieving zero dietary targets with respect to achieving ≥1 (red) or ≥2 (blue) of the targets. Error bars indicate 95% confidence intervals. Lipid measures are from pooled analysis across the 6 time-points and those with skewed distributions were log(x+1)-transformed prior to analyses. C, cholesterol; HDL, high-density lipoprotein; LDL, low-density lipoprotein; VLDL, very-low-density lipoprotein; IDL, intermediate-density lipoprotein.

Figure S3.

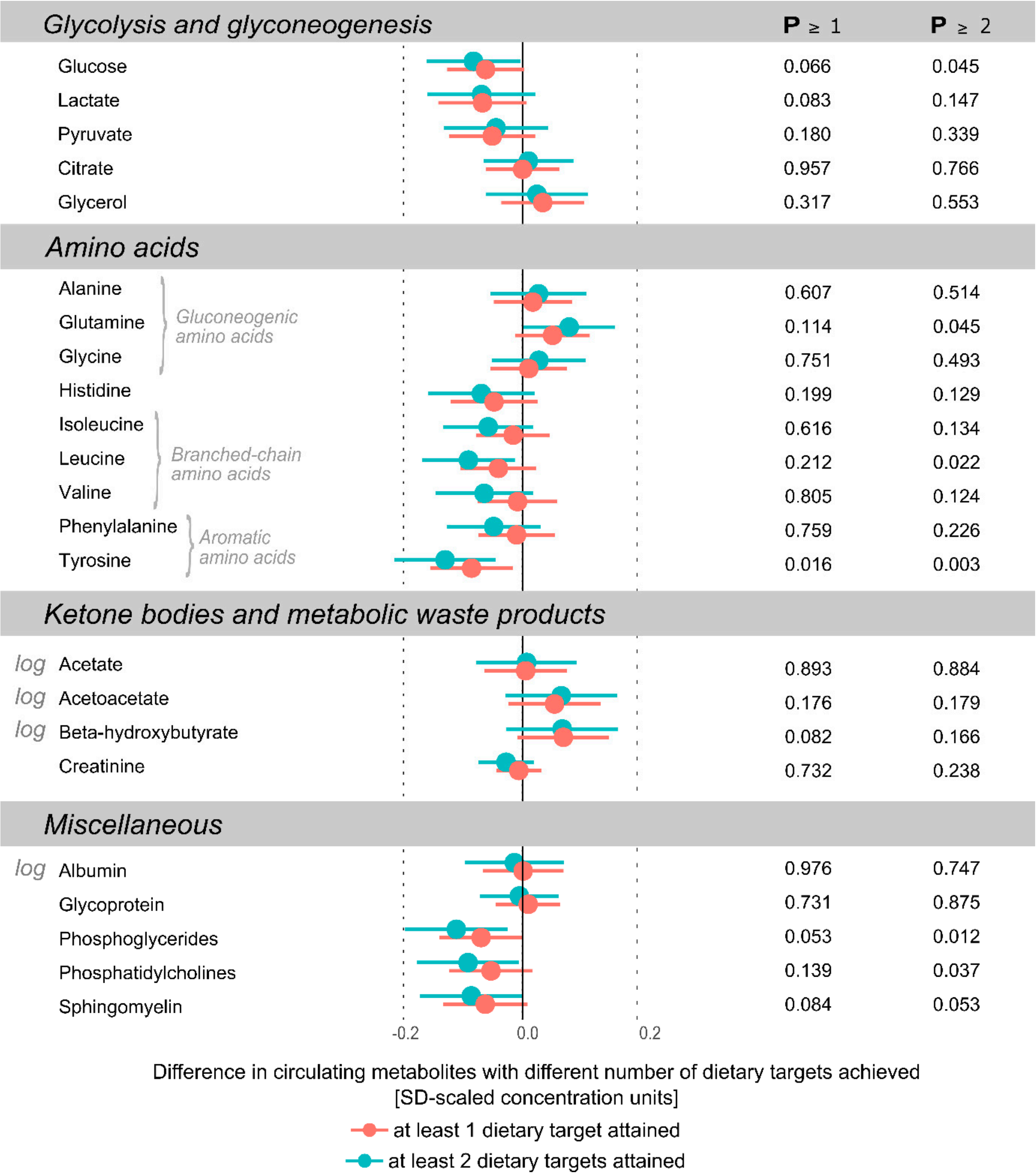

Figure 6. Differences in circulating metabolites between achieving dietary targets compared to achieving none of the dietary targets when dietary target score includes a cholesterol target. Effect estimates are standard deviation scaled differences between achieving zero dietary targets with respect to achieving ≥1 (red) or ≥2 (blue) of the targets. Error bars indicate 95% confidence intervals. Metabolic measures are from pooled analyses across the 6 time-points and those with skewed distributions were log(x+1)-transformed prior to analyses.

Figure S4.

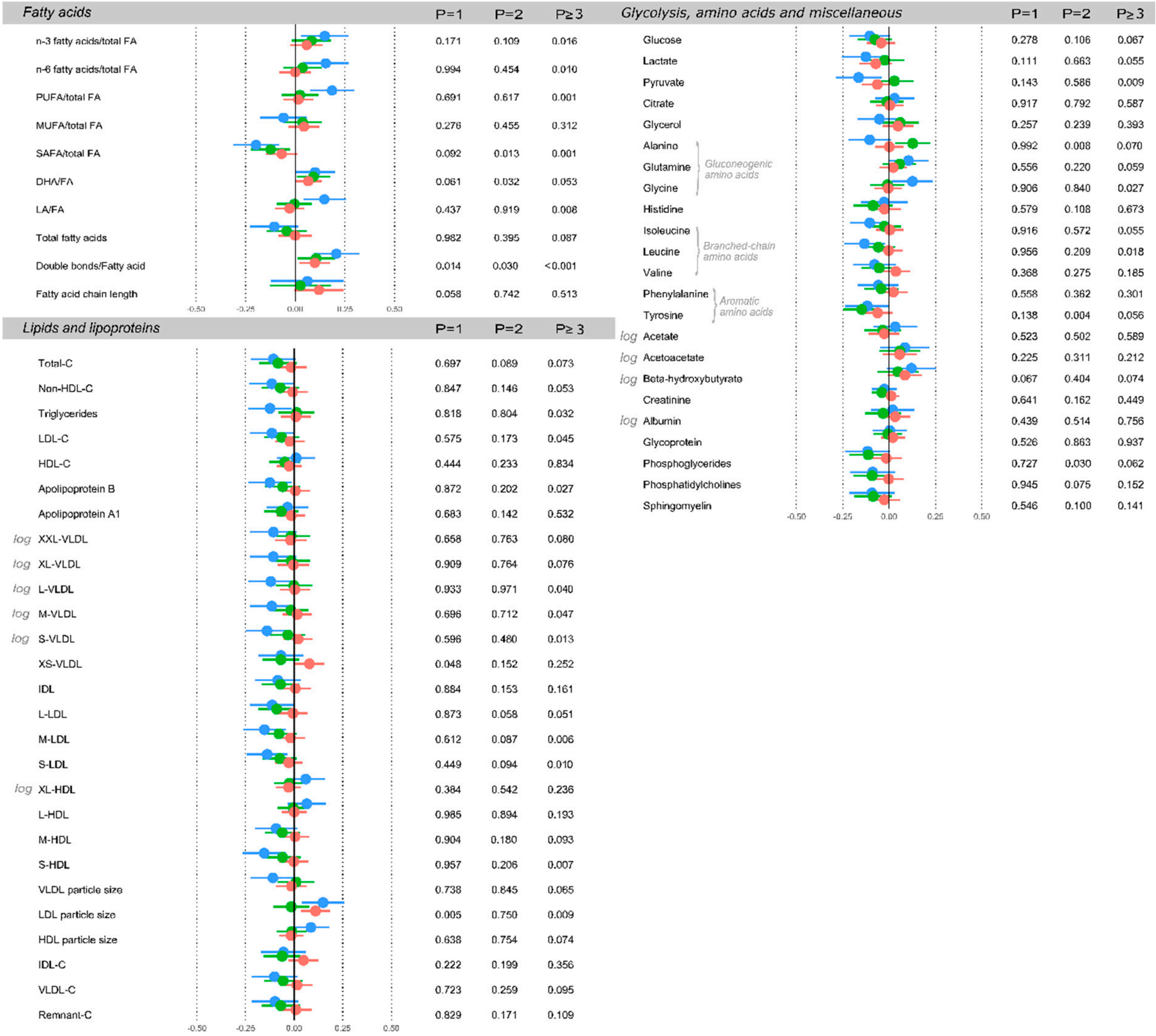

Figure 7. Differences in circulating metabolic measures between achieving dietary targets compared to achieving none of the dietary targets when dietary target score includes a cholesterol target. Effect estimates are standard deviation scaled differences between achieving zero dietary targets with respect to achieving one (red), two (green), or ≥3 (blue) of the targets. Error bars indicate 95% confidence intervals. Metabolic measures are from pooled analyses across the 6 time points and those with skewed distributions were log(x+1)-transformed prior to analyses.

Table S1.

Table 1. Percentage (%) of participants meeting a specific dietary target and percentage (%) of participants achieving 0, ≥1, and ≥2 dietary targets (in parenthesis; corresponding percentage of girls) from 9 to 19 years of age. For fiber and sucrose intake, the value of the corresponding dietary target percentile cut-point is also presented.

Age / Dietary target	9	11	13	15	17	19
S/(P+M) < 1:2	10.0 (47)	12.4 (52)	11.8 (47)	15.0 (45)	17.6 (50)	19.8 (58)
SAFA < 10 E%	20.5 (46)	19.5 (51)	18.4 (57)	25.8 (52)	21.5 (60)	21.3 (57)
Fiber ≥ 80th age-percentile	20.2 (58) 2.34 g/MJ	19.5 (54) 2.28 g/MJ	18.8 (57) 2.26 g/MJ	19.7 (61) 2.46 g/MJ	20.0 (72) 2.64 g/MJ	18.9 (75) 2.65 g/MJ
Sucrose ≤ 20th age-percentile	19.9 (47) 6.7 [E%]	20.5 (42) 6.3 [E%]	20.0 (49) 6.4 [E%]	19.1 (33) 5.7 [E%]	20.0 (44) 5.2 [E%]	20.4 (42) 4.9 [E%]
0 targets achieved	55.6 (51)	53.7 (46)	54.2 (47)	48.7 (46)	50.2 (46)	50.3 (52)
≥1 target achieved	44.4 (48)	46.3 (50)	45.8 (52)	51.3 (48)	49.8 (57)	49.7 (55)
≥2 targets achieved	18.6 (52)	18.9 (47)	16.9 (52)	21.2 (50)	22.2 (56)	21.6 (60)

S/SAFA = Saturated fat; P = polyunsaturated fat; M = monounsaturated fat; MJ = megajoule; E% = percentage of total energy intake

Table S2.

Table 2. Percentage (%) of intervention (I) and control (C) group children meeting a specific dietary target and achieving 0, ≥1, and ≥2 dietary targets from 9 to 19 years of age.

Dietary target \ Age	9		11		13		15		17		19	
	I	C	I	C	I	C	I	C	I	C	I	C
S/(P+M) < 1:2	18	3	25	2	20	5	24	8	24	13	26	15
SAFA < 10 E%	34	9	31	10	28	11	36	18	45	18	28	17
Fiber ≥ 80th age-percentile	28	14	26	14	30	13	23	17	20	20	20	18
Sucrose ≤ 20th age-percentile	23	17	23	18	20	20	18	20	20	20	28	15
0 targets achieved	42	67	40	65	44	63	39	56	47	53	42	56
≥1 target achieved	58	33	60	35	56	37	61	44	53	47	58	44
≥2 targets achieved	31	8	32	8	26	9	27	16	28	18	31	15

S/SAFA = Saturated fat; P = polyunsaturated fat; M = monounsaturated fat; MJ = megajoule; E% = percentage of total energy intake

Table S3.

Table 3. Percentage (%) of participants meeting the additional dietary cholesterol target and percentage (%) of participants achieving 0, ≥1, and ≥2 dietary targets (in parenthesis; corresponding percentage of girls) from 9 to 19 years of age. The dietary cholesterol target percentile cut-point is also presented.

Dietary target \ Age	9	11	13	15	17	19
Cholesterol ≤ 20th age-percentile	20.0 (58) 132 mg	19.9 (58) 137 mg	20.0 (65) 145 mg	19.7 (75) 141 mg	20.5 (87) 133 mg	19.8 (81) 130 mg
0 targets achieved	50.3 (49)	46.1 (42)	47.8 (44)	43.4 (41)	42.4 (38)	44.4 (47)
≥1 target achieved	49.7 (50)	53.9 (53)	52.2 (54)	56.6 (51)	57.6 (61)	55.6 (59)
≥2 targets achieved	24.4 (50)	25.3 (48)	23.5 (56)	28.2 (56)	28.5 (62)	29.3 (66)

Table S4.

Table 4. The mean values for different dietary targets when zero, at least one or at least two dietary targets are achieved.

Mean of dietary target \ Number of targets achieved	0	≥ 1	≥ 2
SAFA/(MUFA+PUFA)	0.41	0.36	0.33
SAFA [E%]	13.6	10.8	9.2
Fiber [g/MJ]	1.7	2.3	2.5
Sucrose [E%]	10.1	7.4	7.2

MJ = megajoule; E% = percentage of total energy intake

Table S5.

Table 5. Effect estimates for all metabolic measures analysed in absolute concentration units when compared to achieving zero dietary targets.

**Metabolite measure [unit]**	**At least one dietary target attained, effect estimate [beta]**	**At least two dietary targets attained, effect estimate [beta]**
ω-3 fatty acids [%]	0.08830	0.15494
ω-6 fatty acids [%]	0.20012	0.36180
PUFA [%]	0.28491	0.47043
MUFA [%]	-0.03896	-0.18101
SAFA [%]	-0.26955	-0.36488
Docosahexaenoic acid [%]	0.05134	0.07639
Linoleic acid [%]	0.09541	0.26496
Total fatty acids [mmol/l]	-0.12002	-0.20510
Degree of unsaturation	0.01029	0.01256
Fatty acid chain length	0.03607	0.01693
Total cholesterol [mmol/l]	-0.06367	-0.11110
Non-HDL cholesterol [mmol/l]	-0.05273	-0.09592

Total triglycerides [mmol/l]	-0.01457	-0.03094
LDL cholesterol [mmol/l]	-0.03269	-0.05508
HDL cholesterol [mmol/l]	-0.01022	-0.01224
Apolipoprotein B [g/l]	-0.01006	-0.01977
Apolipoprotein A-1 [g/l]	-0.01003	-0.01627
log Extremely large VLDL [mmol/l]	-0.00098	-0.00144
log Very large VLDL [mmol/l]	-0.00235	-0.00394
log Large VLDL [mmol/l]	-0.00554	-0.00985
log Medium VLDL [mmol/l]	-0.00644	-0.01176
log Small VLDL [mmol/l]	-0.00420	-0.00938
Very small VLDL [mmol/l]	-0.00108	-0.00935
IDL [mmol/l]	-0.01302	-0.02732
Large LDL [mmol/l]	-0.02239	-0.04405
Medium LDL [mmol/l]	-0.01573	-0.02866
Small LDL [mmol/l]	-0.00970	-0.01667
log Very large HDL [mmol/l]	0.00081	0.00446
Large HDL [mmol/l]	0.00355	0.00532
Medium HDL [mmol/l]	-0.01067	-0.01991
Small HDL [mmol/l]	-0.01097	-0.01816
VLDL particle size (nm)	-0.07017	-0.09244
LDL particle size (nm)	0.01933	0.01995
HDL particle size (nm)	0.00447	0.01028
IDL cholesterol [mmol/l]	-0.00573	-0.01815
VLDL cholesterol [mmol/l]	-0.00961	-0.02028
Remnant cholesterol [mmol/l]	-0.01937	-0.03994
Glucose [mmol/l]	-0.02890	-0.04054
Lactate [mmol/l]	-0.03457	-0.04019
Pyruvate [mmol/l]	-0.00205	-0.00260
Citrate [mmol/l]	0.00031	0.00025
Glycerol [mmol/l]	0.00013	-0.00072
Alanine [mmol/l]	-0.00055	-0.00181
Glutamine [mmol/l]	0.00416	0.00523
Glycine [mmol/l]	-3.18E-06	0.00058
Histidine [mmol/l]	-0.00046	-0.00047
Isoleucine [mmol/l]	-0.00021	-0.00073
Leucine [mmol/l]	-0.00038	-0.00095
Valine [mmol/l]	-9.08E-06	-0.00119
Phenylalanine [mmol/l]	-0.00022	-0.00043
Tyrosine [mmol/l]	-0.00070	-0.00120
log Acetate [mmol/l]	1.39E-05	0.00022
log Acetoacetate [mmol/l]	0.00317	0.00386
log 3-hydroxybutyrate [mmol/l]	0.00421	0.00347
Creatinine [mmol/l]	-0.00015	-0.00037

log Albumin [cu]	-8.59E-05	-0.00026
Glycoprotein acetyls [mmol/l]	-0.00411	-0.00613
Total phosphoglycerides [mmol/l]	-0.03157	-0.05550
Phosphatidylcholines [mmol/l]	-0.02120	-0.04159
Sphingomyelins [mmol/l]	-0.00725	-0.00952

Abbreviations: PUFA, polyunsaturated fatty acids; MUFA, monounsaturated fatty acids; SAFA, saturated fatty acids; HDL, high-density lipoprotein; LDL, low-density lipoprotein; VLDL, very-low-density lipoprotein; IDL, intermediate-density lipoprotein.

Metabolic measures are from pooled analyses across the 6 time-points. 60 metabolic measures were quantified using a high-throughput serum NMR metabolomics platform (6). Quantification of lipoprotein subclasses was calibrated against high performance liquid chromatography on an external set of samples. The 14 lipoprotein subclass sizes were defined as follows: extremely large VLDL with particle diameters from 75 nm upwards and a possible contribution of chylomicrons, five VLDL subclasses (average particle diameters of 64.0 nm, 53.6 nm, 44.5 nm, 36.8 nm, and 31.3 nm), IDL (28.6 nm), three LDL subclasses (25.5 nm, 23.0 nm, and 18.7 nm), and four HDL subclasses (14.3 nm, 12.1 nm, 10.9 nm, and 8.7 nm). The mean size for VLDL, LDL and HDL particles was calculated by weighting the corresponding subclass diameters with their particle concentrations.
